# Verification of a Mobile Psychoacoustic Test System

**DOI:** 10.3390/audiolres11040061

**Published:** 2021-12-13

**Authors:** Jordana C. Soares, Sangamanatha A. Veeranna, Vijay Parsa, Chris Allan, Winnie Ly, Minh Duong, Paula Folkeard, Sheila Moodie, Prudence Allen

**Affiliations:** 1 National Centre for Audiology, Faculty of Health Sciences, Western University, London, ON N6G 1H1, Canada; jordanagri@gmail.com (J.C.S.); s.ankmnalveeranna@usm.edu (S.A.V.); callan3@uwo.ca (C.A.); wingyee.ly@gmail.com (W.L.); mduong29@uwo.ca (M.D.); folkeard@nca.uwo.ca (P.F.); sheila@nca.uwo.ca (S.M.); pallen@uwo.ca (P.A.); 2 School of Speech and Hearing Sciences, The University of Southern Mississippi, Hattiesburg, MS 39401, USA; 3 School of Communication Sciences and Disorders, Western University, London, ON N6A 3K7, Canada; 4 Department of Electrical and Computer Engineering, Western University, London, ON N6A 5B9, Canada

**Keywords:** psychoacoustics, auditory processing, audiology, clinical assessment, tablet computers, system verification, system implementation

## Abstract

Many hearing difficulties can be explained as a loss of audibility, a problem easily detected and treated using standard audiological procedures. Yet, hearing can be much poorer (or more impaired) than audibility predicts because of deficits in the suprathreshold mechanisms that encode the rapidly changing, spectral, temporal, and binaural aspects of the sound. The ability to evaluate these mechanisms requires well-defined stimuli and strict adherence to rigorous psychometric principles. This project reports on the comparison between a laboratory-based and a mobile system’s results for psychoacoustic assessment in adult listeners with normal hearing. A description of both systems employed is provided. Psychoacoustic tests include frequency discrimination, amplitude modulation detection, binaural encoding, and temporal gap detection. Results reported by the mobile system were not significantly different from those collected with the laboratory-based system for most of the tests and were consistent with those reported in the literature. The mobile system has the potential to be a feasible option for the assessment of suprathreshold auditory encoding abilities.

## 1. Introduction

Hearing loss is the third most prevalent chronic disability among older adults and affects an increasing number of younger individuals [1,2]. When unaddressed, hearing loss may impact health, well-being, relationships, communication, education, employment, and finances. Many of the adverse impacts of hearing loss can be mitigated using timely and accurate methods for hearing assessment followed by the implementation of evidence-informed rehabilitative interventions [2].

Audiologic assessment begins with the “audiogram”, an internationally agreed upon metric for reporting hearing loss [3]. Thresholds of sensitivity are measured at discrete frequencies that correspond to the speech range. Test procedures and the reporting of results are standardized allowing comparison of results at different test sites [4,5,6]. However, evaluating only the range of audibility across discrete frequencies fails to capture the hearing difficulties reported by some listeners. For example, twelve percent of adults presenting with hearing difficulties were found to have normal audibility [7].

The discrepancy between hearing threshold levels and hearing performance is especially pronounced when the difficulties arise from neural mechanisms. Disorders of the inner hair cells or the auditory nerve, such as auditory synaptopathy or neuropathy, can significantly reduce understanding with variable impact on threshold level [8]. Additionally, perhaps most clearly the mismatch between audibility and performance can be seen in adults and children with auditory processing disorders [9] for whom difficulty hearing in the presence of normal hearing thresholds is a defining characteristic.

The most common tools for assessing suprathreshold hearing performance are speech tests. Reductions in word discrimination relative to expectations based upon auditory thresholds and difficulty understanding speech that is unfamiliar or degraded by noise, are typically taken to indicate retrocochlear, or hair cell problems [10]. Yet, speech tests are not standardized across practices and performance on speech tests can be confounded by language competency. A child with a language disorder who is referred for auditory processing (AP) testing [11] or an adult being tested using materials in a second language (e.g., [12]) may score poorly on the test for reasons not related to their hearing. For that reason, professional associations recommend that the discrimination of nonspeech sounds should be part of a suprathreshold assessment battery, especially for AP assessment [13,14]. Evaluating the ability of the auditory system to perceive the rapidly changing spectral and temporal characteristics of sound at suprathreshold levels, could shed additional light on hearing difficulties without the confounds of language. Yet, only a few non-speech tests are available to clinicians.

Psychoacoustic assessment can be used to measure the detection and discrimination of suprathreshold features of sounds. Key elements of a psychoacoustic measurement are well-defined stimulus parameters and adherence to strict procedural rules [15]. Laboratory-based studies using psychoacoustic measures have shown that many difficulties experienced by people with hearing complaints beyond that predicted by audibility are attributable to reduced spectral and temporal encoding in the suprathreshold hearing mechanism (e.g., [8,16]). Difficulty discriminating fine differences in frequency, following rapidly changing stimulus envelopes, resolving the temporal fine structure of sounds and many other skills are often impaired in listeners with hearing difficulties. These difficulties are thought to arise from the function of the inner hair cells of the cochlea, the auditory nerve and/or the ascending and descending neural pathways [17,18].

The ability to explore these suprathreshold processes in the clinic could have important implications for differential diagnosis. However, clinicians wishing to administer the psychoacoustic tests for suprathreshold auditory assessment have limited choices. As an example, CD-based psychoacoustic tests require manual set up, administration and scoring, and although modern audiometers may have stimulation generation and processing capabilities required for psychoacoustic testing, they still lack many features for clinical acceptability and efficiency. These important features include automatic delivery of the test regimen, adaptive procedures, automatic scoring, documenting, archiving of test results, and an intuitive user interface. A number of research laboratories have developed customized psychoacoustic tests employing the modular hardware and software to facilitate the implementation of abovementioned features. Unfortunately, the equipment is not easily portable, has a relatively high cost, and generally requires fairly advanced programming skills to operate and evaluate responses. As such, direct clinical adoption of laboratory procedures is not feasible. That can leave much of what is known about hearing processes at suprathreshold levels absent from clinical assessment.

The long-term goal of the project described below is to make laboratory-based procedures for psychoacoustic evaluation available to clinicians. The first step in achieving this goal is the development of a portable, easy to use, tablet-based system that has been evaluated to provide evidence that it can produce the same accuracy in assessment that research-grade equipment does.

## 2. Research Grade/Laboratory Systems for Suprathreshold Measurements

**Figure 1:**
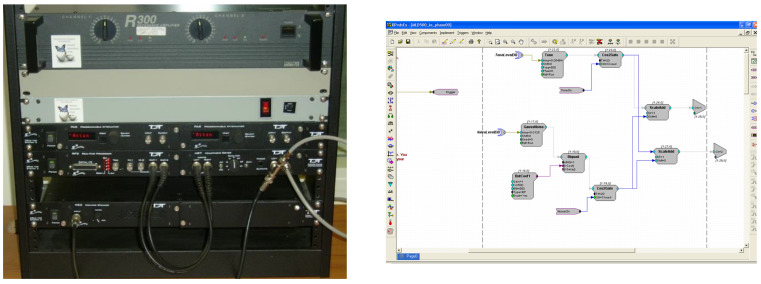
An example of a research-grade laboratory system. The panel on the left shows the equipment rack hosting the real-time signal processing and level control system from Tucker-Davis Technologies, while the right panel depicts a sample custom software “circuit” implementing a psychoacoustic test.

As mentioned above, rigorous and customizable psychoacoustic assessment is most often performed using research-grade laboratory equipment. Through custom software, modified for each listening task, the equipment allows for very detailed specifications of stimulus parameters and computer control of signal delivery, the acquisition of listener responses, and analysis of results. Figure 1 displays an example research-grade system used in our laboratory that is based on the equipment from Tucker-Davis Technologies. The left panel in Figure 1 displays the equipment rack which houses the stimulus generation, level control, and real-time signal processing hardware. The right panel in Figure 1 displays a sample custom software program that implements a specific psychoacoustic test methodology, whose parameters can be programmatically controlled.

In typical psychoacoustic experiments, tasks that assess discrimination abilities most often present listeners with a block of trials in which three sounds are played in sequence. One of the sounds, with equal a priori probability, differs from the other two. The listener’s task is to choose which is different. Most often the procedure is adaptive such that an incorrect response results in a larger difference presented in subsequent trials, while a correct response results in a smaller difference. The adaptive rules can be modified to produce estimates of different performance levels [19]. Trial by trial graphics lead the listener through the series of trials indicating when each sound is played and when a response is required. A progress marker may be used to indicate progression through a block of trials. A strong graphical interface is particularly important when testing children. Colorful animations are commonly used to engage the child in listening and provide feedback on performance accuracy and progress through the block of trials (e.g., [20,21]). The stimulus and animation details are specified in an XML file written for each task, which is then interfaced with the custom software that implements the task, such as the one shown in Figure 1. Our laboratory has used this system extensively to study developmental changes in many complex hearing abilities and how these abilities are affected by hearing disorders [22,23].

## 3. Development of Tablet-Based System

Development of a tablet-based, and therefore more affordable, mobile platform for clinic-based suprathreshold measurements must incorporate a range of auditory function tests, be useful for assessing young children as well as adults, and be verified in advance to provide evidence that test results are equivalent to that obtained in the hearing science laboratory using research-grade laboratory equipment, with similar test rigour.

In the current project, we have endeavored to develop such a mobile system. We based our system development on the SHOEBOX^®^ Audiometry platform—an iPad-based audiometry platform that has been validated for use in a variety of settings [24,25]. This new system and the studies completed to compare it to research-grade laboratory equipment are described in the following sections.

### 3.1. Sound Generation and Delivery

The new system, named the iPad-based Psychoacoustic Assessment System (iPAAS), was built on the sound processing platform at the core of the SHOEBOX^®^ Audiometry applications [24,25]. Salient features of the SHOEBOX^®^ Audiometry sound processing library include (a) calibrated generation and presentation of different signal types (tones, noises, speech, etc.); (b) seamless integration and support for a multitude of transducers; and (c) a patented crosstalk cancellation technique [26] that ensures minimal leakage between the left and right presentation channels in an iPad. For this reason, our group partnered with SHOEBOX^®^ to use their sound libraries to develop the current iPad-based system.

### 3.2. Clinician Interface

**Figure 2:**
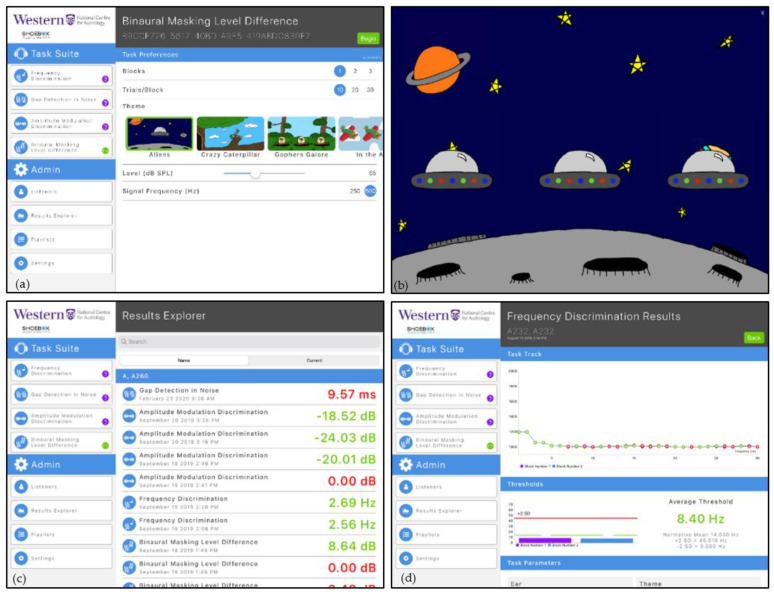
Screenshots from the iPAAS app. (**a**) The main UI where the tester selects the appropriate tests and its parameters; (**b**) cartoon animation during the test; (**c**) color-coded summary of all the test results in the database; and (**d**) trial-by-trial results from a participant for the frequency discrimination test with two blocks.

The iPAAS was developed for use in clinical settings. Because this was an important objective, co-authors with audiological expertise (C.A., S.A.V., J.C.S., and P.A.) provided active input and feedback during the iPAAS development. Figure 2a shows a screenshot of the main user interface. The available psychoacoustic tests are accessible under the “Task Suite” Table. The programmable parameters associated with each test are displayed under the “Task Preferences” Table. The number of trials, number of trial blocks, selected graphic theme, stimulus presentation level and test frequency can be modified by clinicians to select parameters important for their clinical setting and the individual listener/patient receiving care. The “Admin tab” allows access to the listener database (“Listeners” menu item), viewing the details of completed test results (“Results Explorer”), creating and/or activating playlist of planned tests (“Playlists”), and accessing the software and calibration settings (“Settings”).

A playlist for prescribed test selection that includes the task, stimulus and block parameters and number of repetitions can be decided a priori. Once these parameters are set up, the iPAAS system will automatically lead the listener/patient through the evaluation process at their own pace. The reporting of results was designed to include a graphic representation of the trial-by-trial data to aid in the assessment of the patient’s attention to the task and provide an estimate of performance level referenced to age-appropriate standards reported in scientific literature.

### 3.3. Listener/Patient Interface

Because the focus of research in our laboratory is on developmental aspects of hearing and hearing problems in children, colourful graphics were incorporated into the iPAAS software. Key methodological considerations during the iPAAS development included trial by trial guidance through the block of trials without the need for verbal explanations, performance feedback following the trial, and a progress indicator showing progression through the block of trials. Simpler graphics are available for use with adult listeners.

Figure 2b shows an example of the screen that accompanies each trial within the block. The presentation of each signal co-occurs with animation on the screen. Reinforcement is presented for each correctly chosen stimulus. The progress through the test block is indicated on the screen through a separate animation marker that travels left to right.

### 3.4. Patient Performance Data

The Results Explorer, accessible from the “Admin tab” and shown in Figure 2c, shows storage of the estimated thresholds for each of the completed psychoacoustic tasks along with the parameters under which they were measured. To facilitate immediate feedback to the clinicians, the thresholds are coded green if they fall within two standard deviations of the expected mean for that test and red if they do not. Clicking on one of these test results will reveal the trial-by-trial data for that condition. An example is shown in Figure 2d. The y axis shows the value of the dependent variable as a function of trial number on the x axis. Data points are green for correct responses and red for those that were incorrect. These tracks can be used to indicate the reliability of the listener’s responses and give some information about the listener’s attention [21,22]. Thresholds, averaged across blocks, are shown in the bottom portion of this display, along with the comparison to the normative range (mean ± 2 standard deviations). (During the development stages of iPAAS, the normative range for different psychoacoustics tests were taken from the published literature. These normative ranges were updated at the end of the present study to reflect the data collected in this project.) 

## 4. A Side-by-Side Comparison of Research-Grade and Tablet-Based Suprathreshold Measurement Systems

The version of iPAAS described in this report included four tasks: frequency discrimination, amplitude modulation detection, the perception of temporal gaps, and a test of binaural integration. Participants took approximately 8 min to complete two blocks of trials for the frequency discrimination, amplitude modulation detection and gap detection tests. For the binaural masking level difference test, each participant took approximately 8 min (in-phase and out-of-phase condition) to complete one block of trials.

A side-by-side comparison study was conducted to verify that listener performance on the iPAAS was similar to performance on research-grade laboratory equipment for the four tests. A brief description of these four tests, their clinical relevance, their parameters, and testing methodology are described in the following sub-sections.

### 4.1. Frequency Discrimination

The frequency discrimination (FD) task measures the ability to discriminate small differences in frequency [27]. The signal standard frequencies were randomized within a participant. The standard sound is fixed at a predetermined frequency while the frequency of the target (different) stimulus is varied adaptively. A healthy, adult listener can perceive a change in the test frequency of 1 to 2%. Reduced discrimination of frequency differences is often noted in individuals with hearing disorders including sensorineural hearing loss [28], auditory neuropathy [17], and auditory processing disorders [29].

#### Test Parameters: Frequency Discrimination

For the verification process, FD was assessed at standard frequencies of 500, 1000, 2000 and 4000 Hz. FD threshold was determined as the change in signal frequency necessary for detection at an accuracy of 70.7%. Stimuli were 500 ms in duration and separated by a 400 ms inter-stimulus interval. Signals were gated on and off using 10 ms cosine squared ramps. Presentation level of all stimuli was 65 dB SPL.

The frequency of the different signal (target) was adjusted adaptively (see Section 5.4). Two consecutive correct responses resulted in the reduction in the difference between test and target frequencies by a factor of 0.7143. One incorrect response resulted in an increase in the difference between the test and target frequencies by a factor of 1.4 [16].

### 4.2. Amplitude Modulation Detection

The amplitude modulation detection (AMD) test permits examination of the ability of a listener to detect small perturbations in the amplitude of an otherwise steady state, short duration signal. Modulation detection is a critically important aspect of hearing. Slow modulations in continuous discourse signal phrase and word boundaries while slightly faster modulations are used to derive a sound’s temporally changing envelope. Modulation detection is a foundational capability needed for recognizing speech in both quiet and in noisy conditions [30].

To measure modulation detection, a listener is presented with samples of band-limited noise that are either unmodulated or modulated (target). Modulation depth was varied adaptively (see Section 5.4) at a fixed modulation rate. Threshold was taken as the depth of modulation necessary to discriminate between a modulated and unmodulated waveform with 70.7% accuracy at a fixed modulation rate.

Listeners with good hearing usually demonstrate better detection of modulation when the modulation rate is slower [31]. Once the modulation rate exceeds 100 Hz, listeners require a greater depth of modulation for perception. Individuals with hearing difficulties of neural origin require a greater depth of modulation for detection and performance falls off more rapidly as the modulation rate is increased [17,32].

#### Test Parameters: Amplitude Modulation Detection

In this study, the stimulus parameters were set to be consistent with those reported by Hall and Grose [33]. Stimuli were samples of a Gaussian noise with a center frequency of 700 Hz and a bandwidth of 1000 Hz used as the carrier. The duration of the stimuli was 575 ms with an inter-stimulus interval of 400 ms. Modulation detection thresholds were obtained at four modulation rates: 20, 32, 100 and 200 Hz. Modulation rates were randomized within a participant. Modulation depth (*m*) in the target signal could vary between 0 and 1, where 0 represents no modulation and 1.0 signifies 100% modulation depth. The amplitude modulation frequency was held constant, while the modulation depth, *m* was adjusted adaptively, beginning at the modulation depth of 0.75. Two consecutive correct responses or one incorrect response resulted in a change of 0.25 modulation depth. Each subsequent reversal resulted in increase or decrease in the target signal modulation depth by a factor of 0.50. The final step size was 0.01. The average thresholds were converted to dB using 20* log (*m*).

### 4.3. Temporal Gap Detection

The gap detection (GD) task measures the ability to detect a brief interval of silence in an otherwise continuous sample of band-limited, Gaussian noise. It taps a very basic component of temporal acuity [34] that is related to the identification of voicing, the parsing of syllables and the determination of word boundaries [35]. Normal hearing adult listeners can be expected to detect gaps of 3–6 ms [36,37]. Listeners with hearing loss of cochlear origin, auditory neuropathy and auditory processing disorders are less able to perceive brief gaps [17,38]. This may result in a smearing of important temporal information.

#### Test Parameters: Gap Detection

This task required the listener to determine which of three signals contained a brief temporal gap. Details of the method were modeled after those reported previously [21]. Signals were 400 ms samples of Gaussian noise, bandpass filtered centered at 1000 Hz, with a bandwidth of 400 Hz and gated on and off with 10 ms ramps. Samples were separated by a 400 ms inter-stimulus interval. The temporal gaps were gated on and off by a 3 ms ramp. The standard and the target signals were presented at 65 dB SPL. Gap length was varied adaptively (see Section 5.4), beginning at 40 ms. The starting step size was 15 ms. Each subsequent reversal resulted in increase or decrease in the target signal gap length by a factor of 0.50. The final step size factor was 0.25. An uninterrupted, notched noise with a bandwidth of 400 Hz and centered at 1000 Hz, was presented at 25 dB spectrum level (58 dB SPL) to avoid the listener’s use of spectral cues resulting from the fast rise and fall times around the gap [38].

### 4.4. Binaural Release from Masking

Lastly, the binaural masking level difference (BMLD) task measures the sensitivity of the binaural system to interaural differences in-phase [39], an important cue for identifying sound source location and consequently for the ability to segregate sounds originating from different sources [40]. Reduced binaural coding has been reported in patients with sensorineural hearing loss [41,42].

#### Test Parameters: BMLD

In this task, the listener was asked to detect a sample of a binaurally presented 500 Hz pure tone in a 950 Hz wide, Gaussian noise masker centered at 500 Hz. Methodology was modeled after that published by Hall et al. [43]. Both tone and masker were 500 ms in duration with 20 ms cosine squared on and off ramps and separated by a 400 ms inter-stimulus interval. Two conditions were tested with the order randomized between listeners. In one condition the tonal signal and the noise masker were in-phase at the two ears (S_0_N_0_) and in the other the signals were 180° out-of-phase at the two ears but the noise remained in-phase (S_π_N_0_). Thresholds in the out-of-phase condition are expected to be better than in the in-phase condition producing the BMLD. The noise masker was fixed at 65 dB SPL and the tonal signal was varied adaptively beginning at 70 dB SPL. Two consecutive correct responses or one incorrect response resulted in a change of 10 dB in the tonal signal. Each subsequent reversal resulted in an increase or decrease in the target signal by a factor of 0.50. The final step size was 2 dB. The BMLD was derived as the difference between the S_0_N_0_ and S_π_N_0_ threshold.

## 5. System Verification Methodology

To verify that the iPAAS produced equivalent results to that obtained with the laboratory system, these four psychoacoustic tests were measured in normal hearing, adult listeners using both systems. Results from each platform were compared using a within-subjects design.

### 5.1. Participants

This study was approved by the Western University Health Science Research Ethics Board (HSREB), approval No. 102932. Written, informed consent was obtained from all participants before data collection. All data were collected at Western University’s National Centre for Audiology. 

Participants were adults between the ages of 19 and 32 years (FD: *n* = 20, mean age = 26.2 years; GD: *n* = 10, mean age = 24.21 years; AMD: *n* = 10, mean age = 24.4 years; BMLD: *n* = 10 participants, mean age = 25.7 years). Listeners completed each task on both systems. The order of the system tested was systematically counterbalanced between subjects. For each test, half the participants started to be tested with the iPAAS and half started with the research-grade system. Some listeners participated in more than one of the tests but because the tests were run at separate times it was not always possible for each subject to complete all the tests. More specifically, 2 listeners participated in 3 of the 4 tests, and 12 listeners participated in 2 of the 4 tests. 

No participants reported a history of hearing loss, listening complaints, otitis media, academic difficulties, memory deficits or attention complaints. Otoscopic examination showed no obstruction in the external ear canal and no obvious abnormalities of the tympanic membrane. Pure tone detection thresholds were obtained using the GSI-61 clincial audiometer with insert earphones and results were <20 dB HL bilaterally for frequencies at octave intervals from 250 to 8000 Hz [44]. Tympanometry indicated normal middle ear function [45]. Acoustic reflexes with both ipsilateral and contralateral stimulation and recording were also evaluated to assess peripheral integrity, and found to be normal (under 105 dB HL). The presence of distortion product otoacoustic emissions (DPOAE) (≥ 6 dB signal to noise ratio) in both ears, in at least five of eight frequencies suggested normal hearing and healthy cochlear function. DPOAEs were measured using the Vivosonic Integrity (8.8.1.1) and Interacoustics Titan (TM 3.0) measurement systems.

### 5.2. Equipment: The Two Test Systems

#### 5.2.1. Laboratory-Based System

For the verification process, the Tucker-Davis Technologies (TDT) System 3 RP2 real-time signal processor 2.1 was controlled by a Dell Dimension 8100 desktop computer. Each psychoacoustic test was programmed as a software “circuit” (similar to the right panel in Figure 1), and controlled through custom written software. XML files provided experiment details (stimulus characteristics, number of alternatives, adaptive tracking rules, etc.). The samples of tones and or noises were digitally generated with 16 bit resolution and converted to analogue form at a 48,828 Hz sampling rate. Signals were transmitted from the RP2 to a HB-7 headphone amplifier. The signal output was connected, through a patch panel, to the transducers located in the sound booth, where the listener received the test signals. Responses were collected on an Elo Touch system 15” CRT touch monitor model 1525C. Output files included trial by trial records and block level performance estimates.

#### 5.2.2. iPad-Based Assessment System

The iPad-based Psychoacoustic App Suite (iPAAS) was installed on an Apple iPad Pro (64 GB) 10.5”. The sound library module within the iPAAS system digitally generated the samples of tones and/or noises with 16 bit resolution and converted to analogue form at a 44,100 Hz sampling rate. Listener responses were recorded using the iPad touch screen. Individual track data and the final threshold were stored in an internal database.

### 5.3. Signal Presentation and Calibration

All signals were delivered through a transducer placed on the right ear of the listener, except for the BMLD test, in which the stimuli were presented binaurally. The same transducers were used for both systems: Sennheiser HDA 200 circum-aural transducer was used for FD and BMLD tests and the DD-450 circum-aural transducer was for AMD and GD tests. Both transducers have similar frequency response [46].

For both systems, the presentation level of standard and target stimuli was 65 dB SPL. For gap detection, an additional narrowband noise masker was presented at 58 dB SPL, as described in Test Parameters: Gap Detection section. A Brüel and Kjær measuring amplifier Type 2610 and artificial ear Type 4152 were employed for the initial calibration with both the HDA 200 Sennheiser and the DD 450 circum-aural headphones. Additional calibration in FD, BMLD, and GD studies was conducted through the G.R.A.S. Ear Simulator model 43AA with microphone 40AG, and single-channel power supply for preamplifiers G.R.A.S Type 12AK, with the HDA 200 Sennheiser circum-aural headphones. Output levels in all studies were checked through the dual-channel spectrum analyzer Spectra Plus software–SC Sound Card Edition.

**Figure 3:**
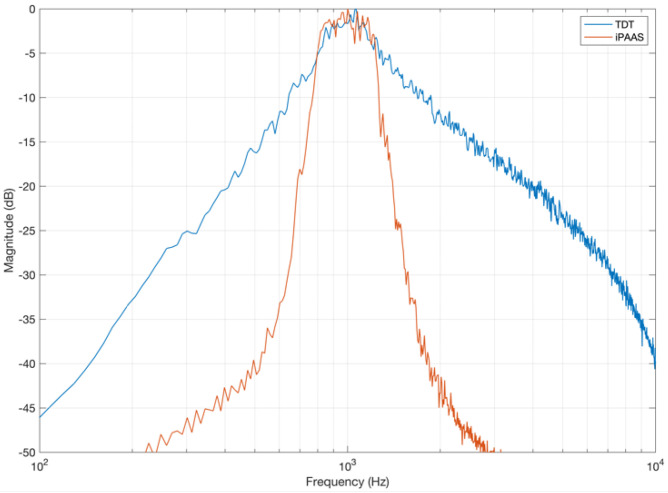
Averaged spectra of the laboratory-based system (TDT) and iPAAS band-limited noise stimuli used for gap detection.

While investigating the stimulus characteristics generated by the two test systems, a difference was observed in the noise stimuli used for the gap detection experiment. For example, the band-limited noise generated by the TDT system had a shallower spectral slope than what was found in the iPAAS (which was significantly steeper), as shown in Figure 3. Similar differences were noted in the narrowband masker, which was included to minimize the perception of any gap-induced spectral splatter. These device-specific differences in the gap stimulus generation can lead to disparate gap detection thresholds, as discussed in the Section 6.

### 5.4. General Procedure

All thresholds were obtained using a three-alternative forced-choice procedure. A two-down one up adaptive procedure [19] tracking 70.7% correct response level was employed. Participants were instructed to listen to three sounds that each played with accompanying cartoon graphics and select the cartoon that sounded different. The interval containing the target signal (the different one) was randomized across the three intervals, with equal a priori probability. On both systems, listeners completed two blocks of 30 trials for each test and condition. For each block, the threshold was calculated by averaging the midpoints between the reversal points. The last four reversal points were used to compute the threshold. The final threshold was calculated by averaging the thresholds from two blocks.

Data were collected and analyzed separately for each test in two different sessions, one day per system. The test order was randomized. During testing the listener was seated comfortably at a small table in the sound isolation booth. To avoid fatigue, participants were encouraged to take breaks during the testing. As noted before, participants took ~16 min to complete two blocks of trials for BMLD, and ~8 min for the other three tasks.

## 6. Results

### 6.1. Frequency Discrimination

**Figure 4:**
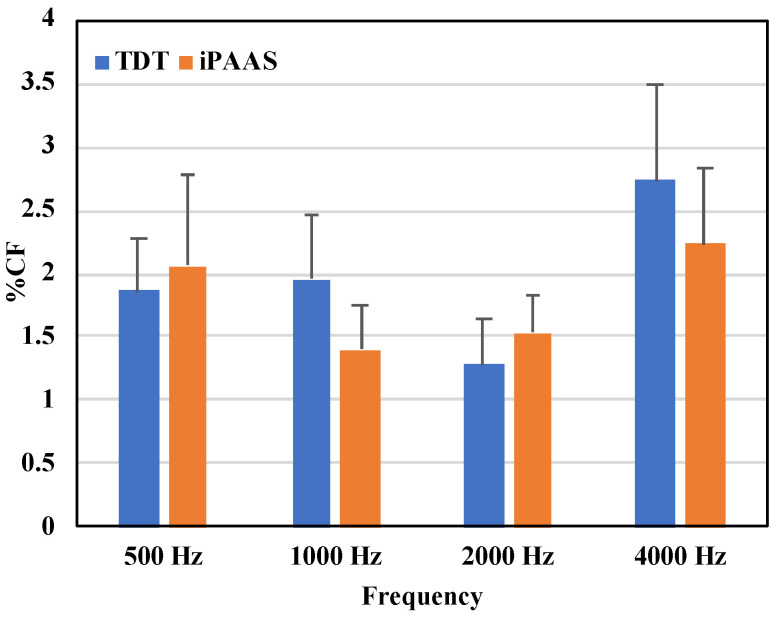
FD thresholds expressed as the proportion of the test center frequency in % for the laboratory-based system (TDT) and iPAAS system. FD thresholds are shown in blue color and orange color for TDT and iPAAS systems, respectively. The error bars represent the standard error of the mean.

FD thresholds in Hz are shown in Table 1, while the same thresholds as a percentage of test center frequency are displayed in Figure 4. The Shapiro–Wilk test of normality showed strong evidence of non-normality in distribution of the data for all frequencies (*p* < 0.05). Threshold values were therefore log transformed, and a repeated measures (RM) ANOVA was performed with the test frequency (500, 1000, 2000, and 4000 Hz), block (first and second block of trials), and system (TDT and iPAAS) as the within-subject factors. The main effect of the system was not significant (F(1,19) = 0.031, *p* = 0.863, $\eta_{p}^{2}=0.002).$ Both the test frequency and the block number were statistically significant (F(3,57) = 124.283, *p* < 0.001, $\eta_{p}^{2}=0.867)$, and (F(1,19) = 9.862, *p* = 0.005, $\eta_{p}^{2}=0.342)$, respectively). None of the higher order interactions were found to be statistically significant. Post hoc Bonferroni tests revealed that the thresholds for 4000 Hz were larger compared to the other test frequencies (*p* < 0.05). In addition, the thresholds associated with the second block were significantly lower compared to those obtained with the first block of trials, for both systems.

Frequency discrimination thresholds obtained with the two systems were therefore equivalent and thresholds were between 1 and 2% of the test frequency at 500, 1000 and 2000 Hz, consistent with previously published results in adults with normal hearing [22,47]. At 4000 Hz thresholds were above 2% in both systems [28].

Overall, results suggest that the tablet-based system provided equally rigorous frequency discrimination thresholds to those reported from laboratory-based studies.

### 6.2. Amplitude Modulation Detection

**Figure 5:**
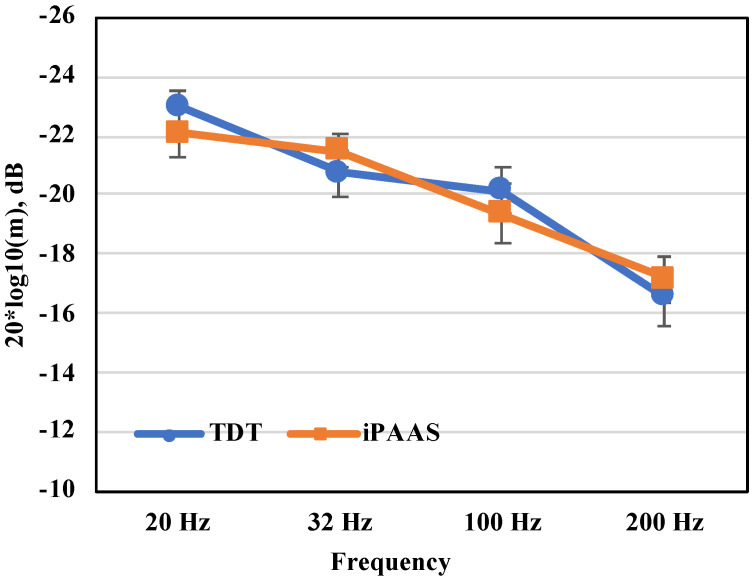
AMD thresholds as a function of modulation frequency. AMD thresholds are shown in filled squares and in filled diamonds for the laboratory-based system (TDT) and iPAAS system, respectively. The error bars represent the standard error of the mean.

The two thresholds estimated at each modulation frequency were averaged. Mean thresholds for modulation detection on the laboratory system and the iPAAS, expressed in dB, are shown in Table 2 and Figure 5. The Shapiro–Wilk test of normality suggested a normal distribution in the mean AM thresholds for both systems (*p* > 0.05). An RM ANOVA was subsequently conducted on the AM threshold data with the modulation frequency (20, 32, 100 and 200 Hz), block (first and second block of trials), and test system (TDT and iPAAS) as the within-subject factors. There were no significant differences in the thresholds estimated from the two platforms (F(1,9) = 0.032, *p* = 0.862, $\eta_{p}^{2}=0.004$) nor was there an effect of the block number (F(1,9) = 1.129, *p* = 0.316, $\eta_{p}^{2}=0.111$). The main effect of frequency was found to be significant (F(3,27) = 25.633, *p* < 0.001, $\eta_{p}^{2}=0.740$). Post hoc testing with the Bonferroni correction showed that the AM thresholds for the 200 Hz modulation frequency were significantly different from those at the other three frequencies (*p* < 0.05).

Overall, the two systems produced equivalent thresholds of modulation detection. Thresholds obtained from both systems showed a low-pass characteristic, i.e., better detection threshold at slow modulation rates when compared to thresholds at faster modulation rates, consistent with the published data [31,32,33].

### 6.3. Gap Detection

Descriptive data for the gap detection thresholds from each system are shown in Table 3. The Shapiro–Wilk test of normality suggested a normal distribution in thresholds for both systems (*p* > 0.05). An RM ANOVA was performed on the gap thresholds with the system (TDT and iPAAS), and the block (first vs. second block of trials) as the within-subject factors. Results showed that the block order was not significant factor (F(1,9) = 3.429, *p* = 0.097, $\eta_{p}^{2}=0.276)$, but there was a significant difference between the thresholds obtained from the two systems (F(1,9) = 49.962, *p* < 0.001, $\eta_{p}^{2}=0.842)$. Gap detection thresholds were lower when measured in the iPAAS system, as can be observed from Table 3.

As discussed in Section 5.3, system differences in the characteristics of stimuli generated for the gap detection test are the potential reason for the discrepancy in gap thresholds. The gap detection thresholds obtained by the iPAAS system are similar to those reported by Lister et al. [36], whose noise spectral characteristics are similar to the iPAAS gap noise spectrum depicted in Figure 3.

### 6.4. Binaural Masking Level Difference

**Figure 6:**
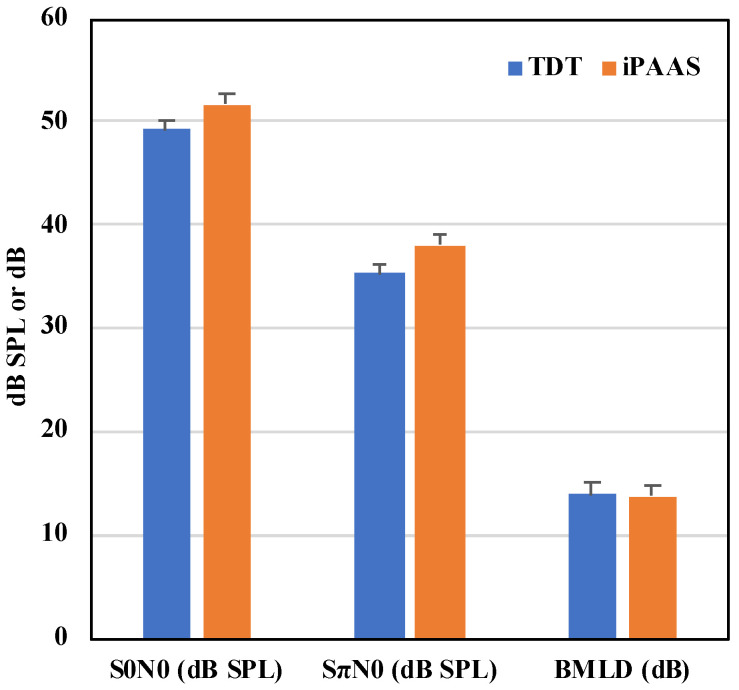
First two sets of bars show the 500 Hz pure tone detection levels in dB SPL in background noise of 70 dB SPL for the in-phase and out-of-phase conditions, respectively. The last set of bars show the BMLD values. The error bars represent the standard error of the mean.

The mean thresholds at the 2 relative phase conditions and their difference (the BMLD) thresholds are shown in Figure 6 and Table 4, respectively. The Shapiro–Wilk test of normality showed that the BMLD thresholds were normally distributed for both systems (*p* > 0.05). In a manner similar to the previous analyses, the RM ANOVA was performed on the BMLD data with the system and block order as the within-subject factors. Neither the system, the block order, nor their interactions were statistically significant (F(1,9) = 0.124, *p* = 0.773, $\eta_{p}^{2}=0.014$; F(1,9) = 1.034, *p* = 0.336, $\eta_{p}^{2}=0.103$; and F(1,9) = 0.001, *p* = 0.982, $\eta_{p}^{2}=0.001,\;\mathrm{respectively}$).

Detection thresholds in both the in- and out-of-phase conditions were equivalent on both systems. Absolute masked thresholds and the difference between the in-phase and out-of-phase conditions, the BMLD, were consistent with those reported in the literature at 500 Hz with normal hearing adult listeners [43].

### 6.5. Bland Altman Analyses

While the RM ANOVA represents a traditional approach for statistical analyses of multiple measurements of the same variable, it may not be an appropriate assessment methodology for measuring the “agreement” between two quantitative measurement methods or systems. Systematic differences between the two measurement methods or systems may not be captured by RM ANOVA [48]. Bland and Altman [48] proposed a methodology where in the differences between the two measurement systems are characterized and the limits of agreement between the two systems are derived. Zou [49] extended Bland–Altman analysis to the data where multiple measurements from each system are available. In this paper, Zou’s method, as implemented in the MedCalc software (v 20.014, MedCalc software, Ostend, Belgium) was utilized to further investigate the agreement between TDT and iPAAS systems.

Within the MedCalc software, the two blocks of repeated measures of FD, AM, BMLD, and GD thresholds were entered for both TDT and iPAAS systems. As mentioned earlier, the FD thresholds were log transformed to satisfy the normality criterion. For each psychoacoustic test, the software calculated the mean of the thresholds estimated by the TDT and iPAAS systems, along with their differences. The bias (i.e., the mean of the differences between the two thresholds) and the limits of agreement (LOA) (±2 standard deviations of the bias) were then calculated and plotted.

**Figure 7:**
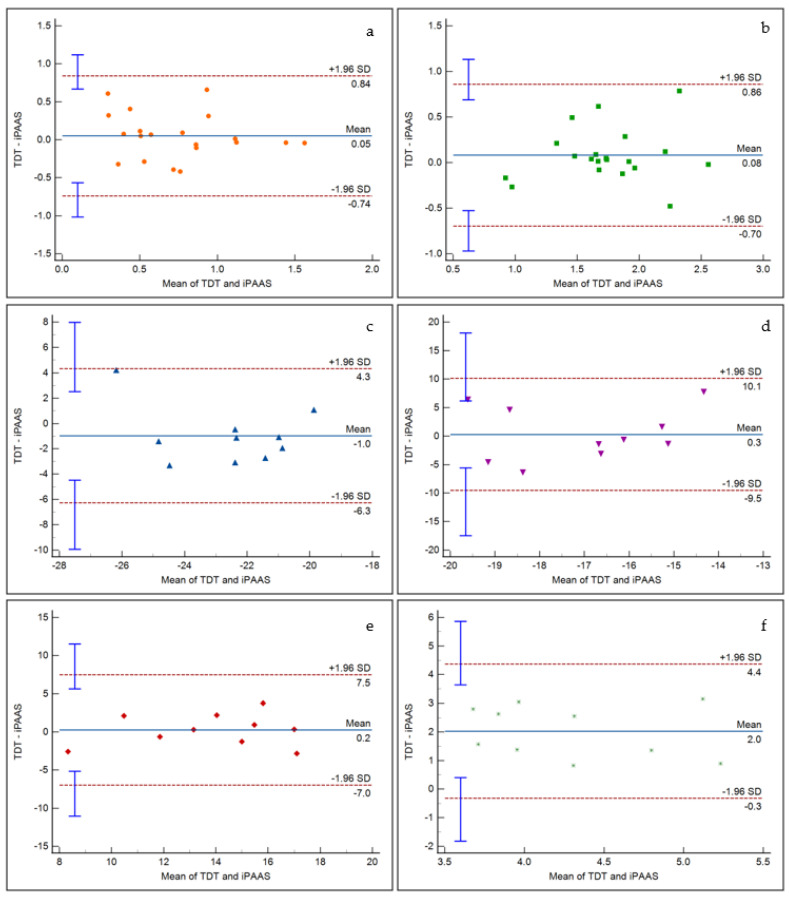
Bland Altman analysis results for (**a**) frequency discrimination at 500 Hz, (**b**) frequency discrimination at 4000 Hz, (**c**) AM detection at 20 Hz modulation frequency, (**d**) AM detection at 200 Hz modulation frequency, (**e**) BMLD at 500 Hz, and (**f**) gap detection. The x axis represents the mean of corresponding TDT and iPAAS thresholds, while the y axis represents their difference. The dashed lines represent the limits of agreements, while the blue error bars represent the corresponding 95% confidence intervals.

Figure 7 displays the representative Bland Altman plots for frequency discrimination at 500 Hz and 4000 Hz, AM detection at 20 and 200 Hz modulation frequencies, BMLD at 500 Hz, and gap detection. It is evident that there is insignificant bias between the two systems for frequency discrimination, AM detection, and BMLD tests. A significant bias did emerge for the gap detection, as noticed in the RM ANOVA results. The scattered data points fit within the 95% confidence intervals for all tests. Furthermore, linear regression fits to the data points resulted in insignificant slope values, indicating that there was no proportional bias in the thresholds estimated by the two systems.

## 7. Discussion

Hearing disorders are common and can be quite debilitating. Although much about hearing loss can be described by threshold elevations as portrayed in the pure-tone audiogram, the ability of the auditory system to encode the rapidly changing spectral, temporal and binaural information in their auditory world is also extremely important. Appreciation of these suprathreshold skills is seldom a part of clinical practice. This paper reports the accuracy of this new behavioral mobile-based system with four psychoacoustic tests and its description. Thresholds from normal hearing adult listeners were obtained and compared to those obtained from an existing laboratory-based system.

The new iPAAS system allows for the collection of laboratory quality psychoacoustic data outside of the laboratory. The iPAAS produced equivalent thresholds to the research-grade system for frequency discrimination, amplitude modulation detection, and binaural masking level differences. System-specific differences in the generation of noise stimuli resulted in consistent difference in the temporal gap detection thresholds between the two systems. This new expandable platform has the potential of providing a portable, affordable and user-friendly tool for assessing suprathreshold spectral, temporal and binaural tasks. Four psychoacoustic tests were included in this study, but the platform has the potential to include other tests.

It must be noted here that other iPad-based psychoacoustic test systems do exist. Feather Squadron is an iPad-based application used to evaluate auditory processing capabilities, specifically designed for children aged 7–14 years [50]. However, the system is constrained to be used with a particular set of headphones (Koss UR10), and the system volume is manually set. The Portable Automated Rapid Testing (PART) is another application designed to examine spectral, temporal, and spatial abilities, using an iPad [51]. The iPAAS incorporates only a subset of psychoacoustic tests available in PART. A further differentiating factor between iPAAS and PART is that the iPAAS incorporates the same stimulus generation and calibration procedures as the SHOEBOX^®^ audiometer, thereby facilitating the assessment of suprathreshold spectral, temporal, and binaural capabilities, in addition to the clinical pure tone audiometry.

### Study Limitations and Future Work

The current study is deemed as a first step in verifying the performance of a new tablet-based psychoacoustic test system. The small sample size (*n* = 10 for three of the four verification studies) can be considered a study limitation. For enabling broader clinical acceptance and uptake, our ongoing and future research studies with the iPAAS focus on: (a) the collection and refinement of age-appropriate normative data from a greater number of normal hearing adults and children; (b) collection of psychoacoustic test data from adults and children with sensorineural hearing loss; and (c) collection of psychoacoustic test data from children diagnosed with suspected central auditory processing disorder.

The iPAAS development used a knowledge translation approach [52], where feedback from audiologists’ in clinical practice was used to refine the user interface, a user’s guide, and a video user’s guide. However, the iPAAS has not yet been field-tested in a standard clinical setting, nor feedback from practicing clinical audiologists on this new tool’s utility and functionality has been garnered. Further research is therefore warranted on obtaining clinicians’ opinions regarding the iPAAS test platform’s usability, utility, feasibility for clinical practice, as well as assisting us with understanding the barriers and facilitators to routine clinical implementation of psychoacoustic testing [53].

## 8. Conclusions

This paper describes the collaborative development of a tablet-based system (the iPAAS) that can be used in clinical practice for suprathreshold temporal, spectral, and binaural auditory capabilities. This new tool is innovative and important as it brings additional possibilities for broader testing of listening abilities with more affordable, clinically usable equipment. The iPAAS produced equivalent thresholds to the research-grade system for frequency discrimination, amplitude modulation detection, and binaural masking level differences. System-specific differences in the generation of noise stimuli resulted in consistent difference in the temporal gap detection thresholds between the two systems.

## Figures and Tables

**Table 1:** Descriptive results for the FD thresholds (given in Hertz) for the laboratory-based system (TDT) and iPAAS system.

	500 Hz		1000 Hz		2000 Hz		4000 Hz	
	TDT	iPAAS	TDT	iPAAS	TDT	iPAAS	TDT	iPAAS
Mean	9.3	10.3	19.6	14.0	25.7	30.6	109.6	89.2
SD	9.2	15.9	23.1	15.5	33.1	26.1	136.2	107.3
Mean + 2 SD	27.7	42.1	65.8	45.0	91.9	82.7	382.0	303.8
Minimum	1.7	1.2	1.9	2.9	3.2	7.9	6.9	10.2
Maximum	35.5	71.9	97.2	58.4	148.9	104.7	523.6	439.8

**Table 2:** Descriptive results for the AMD thresholds (given in dB) for the laboratory-based system (TDT) and iPAAS system.

	20 Hz		32 Hz		100 Hz		200 Hz	
	TDT	iPAAS	TDT	iPAAS	TDT	iPAAS	TDT	iPAAS
Mean	−23.1	−22.1	−21.1	−21.5	−19.6	−19.4	−16.84	−17.2
SD	2.0	2.6	2.5	2.0	1.9	3.3	3.22	2.7
Mean + 2 SD	−19.1	−16.9	−15.5	−17.6	−15.0	−12.7	−10.19	−11.7
Minimum	−19.3	−28.3	−16.0	−23.4	−15.8	−25.7	−10.40	−22.8
Maximum	−26.1	−19.9	−24.5	−18.5	−21.8	−14.6	−21.54	−14.5

**Table 3:** Descriptive results for the GD thresholds (given in ms) for the laboratory-based system (TDT) and iPAAS system.

	TDT	iPAAS
Mean	5.2	3.3
SD	0.7	0.8
Mean + 2 SD	6.6	4.9
Minimum	4.5	2.3
Maximum	6.7	4.8

**Table 4:** Descriptive results for the BMLD thresholds (given in dB) for the laboratory-based system (TDT) and iPAAS system.

	TDT	iPAAS
Mean	13.9	13.7
SD	3.2	2.3
Mean − 2 SD	7.5	7.9
Minimum	7.0	9.4
Maximum	17.7	18.5

## Data Availability

The data presented in this study are available on request from the corresponding author.
